# POMC Neuron BBSome Regulation of Body Weight is Independent of its Ciliary Function

**DOI:** 10.1093/function/zqad070

**Published:** 2023-12-23

**Authors:** Deng-Fu Guo, Paul A Williams, Connor Laule, Charles Seaby, Qihong Zhang, Val C Sheffield, Kamal Rahmouni

**Affiliations:** Department of Neuroscience and Pharmacology, University of Iowa Carver College of Medicine, Iowa City, IA 52242, USA; Veterans Affairs Health Care System, Iowa City, IA 52242, USA; Department of Neuroscience and Pharmacology, University of Iowa Carver College of Medicine, Iowa City, IA 52242, USA; Department of Neuroscience and Pharmacology, University of Iowa Carver College of Medicine, Iowa City, IA 52242, USA; Department of Pediatrics, University of Iowa Carver College of Medicine, Iowa City, IA 52242, USA; Department of Pediatrics, University of Iowa Carver College of Medicine, Iowa City, IA 52242, USA; Department of Pediatrics, University of Iowa Carver College of Medicine, Iowa City, IA 52242, USA; Fraternal Order of Eagles Diabetes Research Center, University of Iowa Carver College of Medicine, Iowa City, IA 52242, USA; Iowa Neuroscience Institute, University of Iowa Carver College of Medicine, Iowa City, IA 52242, USA; Department of Neuroscience and Pharmacology, University of Iowa Carver College of Medicine, Iowa City, IA 52242, USA; Veterans Affairs Health Care System, Iowa City, IA 52242, USA; Fraternal Order of Eagles Diabetes Research Center, University of Iowa Carver College of Medicine, Iowa City, IA 52242, USA; Iowa Neuroscience Institute, University of Iowa Carver College of Medicine, Iowa City, IA 52242, USA; Obesity Research and Education Initiative, University of Iowa Carver College of Medicine, Iowa City, IA 52242, USA; Department of Internal Medicine, University of Iowa Carver College of Medicine, Iowa City, IA 52242, USA

**Keywords:** Bardet-Biedl syndrome proteins, hypothalamus, proopiomelanocortin neurons, energy balance

## Abstract

The BBSome, a complex of several Bardet-Biedl syndrome (BBS) proteins including BBS1, has emerged as a critical regulator of energy homeostasis. Although the BBSome is best known for its involvement in cilia trafficking, through a process that involve BBS3, it also regulates the localization of cell membrane receptors underlying metabolic regulation. Here, we show that inducible *Bbs1* gene deletion selectively in proopiomelanocortin (POMC) neurons cause a gradual increase in body weight, which was associated with higher fat mass. In contrast, inducible deletion of *Bbs3* gene in POMC neurons failed to affect body weight and adiposity. Interestingly, loss of BBS1 in POMC neurons led to glucose intolerance and insulin insensitivity, whereas BBS3 deficiency in these neurons is associated with slight impairment in glucose handling, but normal insulin sensitivity. BBS1 deficiency altered the plasma membrane localization of serotonin 5-HT2C receptor (5-HT_2C_R) and ciliary trafficking of neuropeptide Y2 receptor (NPY_2_R).In contrast, BBS3 deficiency, which disrupted the ciliary localization of the BBSome, did not interfere with plasma membrane expression of 5-HT_2C_R, but reduced the trafficking of NPY_2_R to cilia. We also show that deficiency in BBS1, but not BBS3, alters mitochondria dynamics and decreased total and phosphorylated levels of dynamin-like protein 1 (DRP1) protein. Importantly, rescuing DRP1 activity restored mitochondria dynamics and localization of 5-HT_2C_R and NPY_2_R in BBS1-deficient cells. The contrasting effects on energy and glucose homeostasis evoked by POMC neuron deletion of BBS1 versus BBS3 indicate that BBSome regulation of metabolism is not related to its ciliary function in these neurons.

## Graphical Abstract

**Graphical Abstract:**
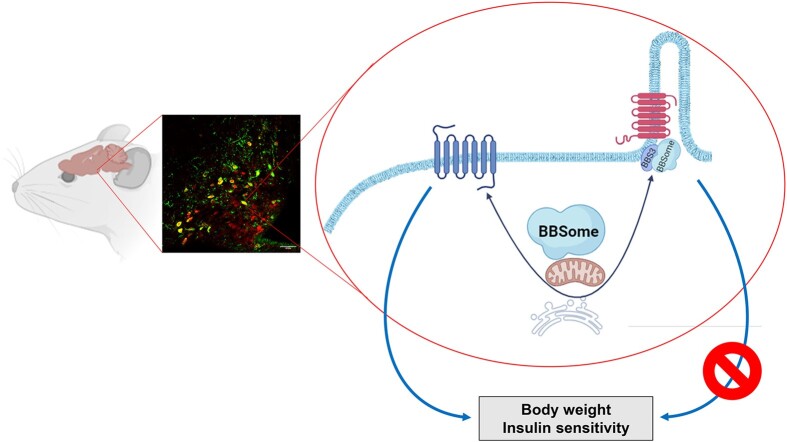

## Introduction

The high prevalence of obesity is a serious health hazard and represents a growing public health concern.^1–3^ Strong evidence points to the central nervous system in obesity susceptibility,^4, 5^ which is consistent with the critical role of the brain, particularly the hypothalamus, in the regulation of body weight by integrating the external and internal signals and enacting appropriate and consequential metabolic and behavioral responses to maintain energy balance.^6, 7^ Proopiomelanocortin (POMC) neurons are essential for energy homeostasis due to their importance in mediating the effects of various signals.^8^ Proopiomelanocortin products such as α-melanocyte stimulating hormone, which activates the melanocortin 4 receptor (MC4R), have potent and long-lasting anorectic and weight-reducing effects. While several molecules in POMC neurons have been identified as playing a key role in the control of energy homeostasis, our understanding of the molecular basis of metabolic regulation by POMC neurons remain incomplete.

Bardet-Biedl syndrome (BBS) proteins have emerged as key components in metabolic control.^9^ The requirement of BBS proteins for metabolic homeostasis is supported by the high prevalence of obesity and other metabolic disturbances including type 2 diabetes in individuals that lack functional BBS proteins.^10, 11^ A total of 8 conserved BBS proteins (BBS1, BBS2, BBS4, BBS5, BBS7, BBS8, BBS9, and BBS18) interact together to form a complex, termed BBSome.^12^ BBSome assembly relies on another protein complex, referred to as BBS chaperonin complex, which contains 3 BBS proteins (BBS6, BBS10, and BBS12).^13^ The BBSome is best known for its critical role in the regulation of cilia function by transporting cargos in and out of the cilium, an organelle that is present in neurons throughout the central nervous system including POMC neurons.^14–16^ For instance, the BBSome was found to mediate the ciliary localization of receptors implicated in metabolic regulation such as the neuropeptide Y2 receptor (NPY_2_R).^17^ Such findings have led to the notion that BBS-associated obesity is caused by loss of ciliary localization of these receptors.^18, 19^ However, the BBSome has been implicated in many cellular processes not related to cilia including the delivery to the plasma membrane of receptors involved in the regulation of energy homeostasis such as the serotonin 5-HT2C receptor (5-HT_2C_R).^16, 20, 21^ More recently, we demonstrated the importance of the BBSome for the regulation of mitochondria function by modulating the activity of dynamin-like protein 1 (DRP1).^22^

BBS3 (also known as ARL6), a member of the Arf subfamily of the Ras superfamily of small GTPases, controls BBSome recruitment to the membrane and its ciliary entry in a GTP-dependent manner.^23–25^ Thus, loss of BBS3 interrupt the ciliary localization of the BBSome. Interestingly, *Bbs3* gene null mice do not phenocopy many of the features displayed by BBSome-deficient mice.^26^ In particular, *Bbs3* null mice exhibited a modest increase in body weight and fat mass whereas mice lacking elements of the BBSome display overt obesity and associated diseases including type 2 diabetes.^11, 21, 27, 28^ These results suggest that BBSome regulation of body weight and energy homeostasis is not related to its function in cilia. In the current study, we used in vivo and in vitro systems to assess the effects of disrupting ciliary localization of the BBSome through deletion of BBS3 compared to disruption of the complex itself through BBS1 deletion. For the in vivo studies, we elected to target POMC neurons since the obesity phenotype was largely recapitulated by disruption of the BBSome, though constitutive *Bbs1* gene ablation, in these neurons.^16^ The use of inducible POMC^Cre^ mouse model allowed us to determine whether the obesity phenotype evoked by congenital BBSome deficiency is recapitulated by the disruption of this complex in adult stage.

## Materials and Methods

### Cell Culture and Constructs

We used a CRISPR-Cas9 system to produce cell lines lacking *BBS1* or *BBS3* gene. Specifically, pSpCas9-BBS1g2 and pSpCas9-BBS3g2 were used to generate the retinal pigment epithelial RPE1-*BBS1^−/−^* and RPE1-*BBS3^−/−^* cells, respectively. The guide sequence used for *BBS1* is CCTTTGAGCACCTTCAGGCG and for *BBS3* is GTGTTTGACATGTCAGGTCA. Stable clones were selected with blasticidine and indels were identified by Sanger sequencing. It should be noted that wild-type RPE1 cells derived from the same clones as the null cells were used as controls. Stable clones were also verified by Western blot. Human NPY_2_R and 5HT_2C_R were tagged with a green fluorescent protein (GFP) at C-terminus as descripted previously.^16^

### Antibodies

Antibodies used in this study consist of those targeting BBS3 (Santa Cruz Biotechnology, Cat# sc-390021), BBS2 (Santa Cruz Biotechnology, Cat# sc-365355 and Proteintech, Cat# 11188-2-AP), acetylated α-tubulin (Santa Cruz Biotechnology, Cat# sc-23950), ADP ribosylation factor like GTPase 13B (ARL13B, Proteintech, Cat# 17711-1-AP), β-actin (Proteintech, Cat# 66009-1-Ig), NPY_2_R (Neuromics, Cat# RA14112), POMC (Phoenix Pharmaceuticals, INC, Cat# H-029-30), DRP1 (BD Transduction Laboratories, Cat# 611113), and phospho-DRP1 (Ser616, Cell Signaling, Cat# 3455). For Western blotting, primary antibodies were diluted 1:1000 in 5% bovine serum albumin (BSA). For immunofluorescent staining, primary antibodies were diluted 1:250.

### Animals

All animal testing was performed based on guidelines set forth by the National Institutes of Health and approved by the University of Iowa Animal Care and Use Committee. Mice were housed in groups of 3-5 per cage and maintained on 12-h light-dark cycle with lights on at 6 am. Room temperature was maintained at 22°C. Food and water were available ad libitum except when the mice were fasted as indicated below.

Bbs1^fl/fl^ mice that carry floxed alleles around exon 3 of the *Bbs1* gene were generated previously.^29^ Bbs3^fl/fl^ mice were generated by inserting LoxP sites around exon 5 of the *Bbs3* gene using an EuMMCR targeting vector (PG00267_Y_7_A07). Cre-recombinase leads to the excision of exon 5. Female Bbs1^fl/fl^ and Bbs3^fl/fl^ mice were crossed with POMC^CreERT2^ mice^30^ (provided by Dr. Joel Elmquist, UT Southwestern) to generate POMC^CreERT2^/Bbs1^fl/fl^ and POMC^CreERT2^/Bbs3^fl/fl^ mice, respectively. Littermate Bbs1^fl/fl^ and Bbs3^fl/fl^ mice were used as controls. To label POMC neurons, POMC^CreERT2^/Bbs1^fl/fl^ and POMC^CreERT2^/Bbs3^fl/fl^ mice were further crossed with the ROSA (Stop^fl/fl^-tdTomato) reporter transgenic mice (Jackson Laboratory, strain numbers 007914). Cre recombination removes the stop site, leading to the expression of the fluorescent tdTomato protein in POMC neurons.

The genotype of the mice was determined by polymerase chain reaction (PCR) analysis of tail DNA using the following conditions: 95°C for 30 s, 60°C for 30 s, and 72°C for 30 s for a total of 34 cycles. Primers used for genotyping are provided in the Table S1. To induce Cre recombinase expression, 6-wk-old POMC^CreERT2^/Bbs1^fl/fl^ and POMC^CreERT2^/Bbs3^fl/fl^ mice and their littermate controls (Bbs1^fl/fl^ and Bbs3^fl/fl^ mice) were administrated with 75 mg/kg of tamoxifen for 5 d via intraperitoneal (IP) injection. In the body weight study, a small subset of male POMC^CreERT2^/Bbs1^fl/fl^ mice (*n* = 4) treated with vehicle (corn oil) was used as an additional control.

### Validation of Deletion of *Bbs1* and *Bbs3* Gene in POMC Neurons

Single cell RNA PCR was used to verify selective *Bbs1* and *Bbs3* gene deletion in POMC neurons of the hypothalamic arcuate nucleus. A 3-mo-old male POMC^CreERT2^/Bbs1^fl/fl^/ROSA and POMC^CreERT2^/Bbs3^fl/fl^/ROSA mice were intraperitoneally injected with tamoxifen (75 mg/kg) for 5 consecutive days. Ten days after the first tamoxifen injection, mice were sacrificed and perfused with a cold cutting solution containing (in m m) 92 NMDG, 2.5 KCl, 1.2 NaH_2_PO_4_, 30 NaHCO_3_, 20 HEPES, 25 glucose, 5 sodium ascorbate, 2 thiourea, 3 sodium pyruvate, 10 MgSO_4_, and 0.5 CaCl_2_. Retrieved brains were submerged in a chilled and aerated (95% O_2_, 5% CO_2_) cutting solution before sections (280 µm) containing the hypothalamic arcuate nucleus were obtained using a vibratome. Brain slices were transferred to an aerated and heated (33°C) bath containing the same solution for 15 min. Sections were then incubated at room temperature for 45 min in a holding solution containing (in m m) 92 NaCl, 2.5 KCl, 1.2 NaH_2_PO_4_, 30 NaHCO_3_, 20 HEPES, 25 glucose, 5 sodium ascorbate, 2 thiourea, 3 sodium pyruvate, 2 MgSO_4_, and 2 CaCl_2_. Slices were next placed in a recording chamber perfused with aerated artificial cerebral spinal fluid containing (in m m) 124 NaCl, 2.5 KCl, 1.2 NaH_2_PO_4_, 24 NaHCO_3_, 5 HEPES, 12.5 glucose, 2 MgSO_4_, and 2 CaCl_2_. GΩ seals on arcuate tdTomato^+^ or tdTomato^−^ cells were made with an electrophysiological recording pipette and removed from the brain slice. Pipette tips containing a single neuron were broken off into RNA extraction buffer and further processed for single cell PCR for *Bbs1, Bbs3, Pomc*, and *S18* genes according to the manufacture’s protocol (PicoPure RNA Isolation Kit, Thermo Fisher). Ribonucleic acid was collected with 16 μL elution buffer, then reverse transcription were performed in total of 20 μL with Maxima H Minus cDNA Synthesis Master Mix Kit from Thermo Scientific according to their instruction. Polymerase chain reaction was performed as 2 steps with 1.5 μL primers, 5 μL cDNA, and 20 μL of Platinum Hot Start PCR 2X Mater Mix from Invitrogen as 94°C for 5 min, 34 cycles for 94°C, 30 s, 58°C for 30 s, and 72°C for 30 s, then 72°C for 3 min. The second step was repeated same as first step except 5 μL of first step PCR product instead of cDNA. The final PCR products were separated in 2% agarose gel and image was taken under UV light. Primer sequences are provided in the Table S1.

### Analysis of Body Weight, Adiposity, and Food Intake

Body weight analysis was performed by weighing mice once a week for 20 wk, from weaning. Nuclear magnetic resonance (NMR, LF50, Bruker minispec) was used to measure body composition (fat mass and lean mass) in mice. At the end of experiments, mice were sacrificed and various fat pads (interscapular brown, perirenal, gonadal, and inguinal fat pads), liver, and kidneys were dissected and weighed. To measure food intake, mice were housed in individual cages. After 3 d of acclimation to individual housing, daily and cumulative food intake were measured over a 4-d period at 20 wk of age.

### Glucose and Insulin Tolerance Tests

Glucose tolerance test was performed in overnight fasted mice. Blood samples were taken from the tail to measure blood glucose at baseline before mice were injected with glucose (2 mg/kg body weight, IP, Sigma-Aldrich). To test for the glucose-reducing effect of insulin, mice were fasted for 5 h. After determining baseline glucose levels, mice were injected with insulin (0.5 unit/kg body weight, IP, Novo Nordisk). Blood glucose was measured at 15, 30, 60, and 120 min after injection of glucose or insulin. Blood glucose levels were determined using a glucometer (OneTouch Ultra 1, LifeScan Inc.).

### Cell Transfection, Infection, and Immunofluorescence

RPE1, RPE1-*BBS1^−/−^*, and RPE1-*BBS3^−/−^* cells were gown in DMEM/F12 medium. The cells were seeded in 24 wells with glass cover slide the day before transfection. Density of the cells was 95% confluent in order to promote cilia formation at transfection. Lipofectamine 3000 transfection reagent was used according to manufacture’s instructions. Forty hours post transfection, the cells were serum starved overnight. RPE1-*BBS1^−/−^* cells infected with an adeno-associated virus 2/5 expressing the mouse *Bbs1* gene (AAV-*Bbs1*, ∼100 particles/cell) for 48 h were also subject to overnight serum starvation. The cells were then fixed with 4% paraformaldehyde (PFA) for 20 min in room temperature, blocked with blocking solution containing 5% normal goat serum and 0.1% Triton X-100 in phosphate-buffered saline (PBS) before incubation with the primary antibody (1:250), at 4°C overnight. The cells were washed with PBS for 10 min, 3 times followed by incubation with secondary antibody, diluted at 1:2000, in room temperature for 1 h. The cells were washed again with PBS for 10 min, 3 times before there were mounted with VECTASHIELD mounting solution with DAPI (4′,6-diamidino-2-phenylindole) that stain cell nuclei.

To test whether rescue of mitochondria dynamic restore localization of 5HT_2C_R to the plasma membrane and NPY_2_R to the cilia, RPE1 and RPE1-*BBS1^−/−^* cells were transiently transfected with 5HT_2C_R-YFP or NPY_2_R-GFP. After 6 h transfection, the cells were infected with FIV-DRP1S637A virus (∼100 particles/cell) for 48 h. It is worth noting that the FIV-DRP1S637A expresses an shRNA that suppresses endogenous DRP1 expression. Cells were then processed for cilia staining with the ARL13B antibody as above.

All images were captured using confocal microscopy (Zeiss LSM880). We counted the number of cilia that are NPY_2_R-positive or -negative in each image before averaging the data for each replicate.

### Brain Slice Immunostaining

Mice deeply anesthetized with ketamine and xylazine were perfused with PBS (3 mL/min; 15 mL) followed by 4% PFA/HistoChoice Tissue Fixative (Amresco) in PBS (3 mL/min; 45 mL) using Harvard PHD 22/2000 Syringe Pump. Entire brain was extracted and incubated in the same fixative overnight at 4°C. Fixed brains were washed 3 times with PBS and incubated in 30% sucrose/PBS overnight with 1 change of solution after 4-6 h of initial incubation. Brains were vibratome-sectioned with 30 μm thickness. Immunostaining was performed on brain sections to detect POMC or NPY_2_R as described previously^20^ by using a 1:250 dilution of a rabbit polyclonal anti-POMC or -NPY_2_R antibodies. Processed brain sections were mounted using VECTASHIELD mounting medium with DAPI. For each animal, NPY_2_R co-colocalization with cilia was quantified in multiple confocal images (Zeiss LSM880) before calculating the average for each mouse.

### Mitochondrial-targeted GFP

Mitochondrial morphology was analyzed using mitochondrial-targeted GFP (mito-GFP) as described before.^22^ Briefly, cells grown on coverslips precoated with 0.1% gelatin were transduced with adenovirus expressing mito-GFP (MOI 50) for 48 h, fixed in 4% paraformaldehyde and then mounted in VECTASHIELD mounting medium with 4,6-diamidino-2-phenylindole (DAPI). Images were acquired using a Zeiss LAM 880 confocal microscope. National Institutes of Health ImageJ was used for morphometry analysis of length and form factor (length/width) as previously reported.^22^ The parameters for form factor are set with minimum value of 1 for perfectly circular mitochondria.

### Mitochondrial Morphometry by Transmission Electron Microscopy

Cells were fixed with 2.5% glutaraldehyde (electron microscopy grade) and 2% paraformaldehyde in PBS and then washed with ice-cold 0.1 m sodium cacodylate 3 × 10 min on ice followed by postfixation in 1% osmium tetroxide, 0.8% potassium ferrocyanide in 0.1 m sodium cacodylate for 3 h on ice. After 3 washes in ice-cold ddH_2_O for 10 min each, cells were stained in 2% uranyl acetate for 2 h before dehydration in an ethanol series of ice-cold 20%, 50%, 70%, and 90%, followed by 3 washes in 100% ethanol at room temperature for 10 min each. Samples were infiltrated in 67% ethanol/33% Durcupan ACM (Fluka; Sigma-Aldrich) for 3 h at room temperature with agitation, then 33% ethanol/67% Durcupan ACM for 3 h at room temperature followed by 3 changes of 100% Durcupan for 8 h each at 22°C with agitation. The Durcupan-infiltrated cells were then flat-mounted between 2 mold-release glass slides and polymerized at 60°C for 2 d. Semithick sections were cut using a Leica EM UC7 ultramicrotome and placed on 50-mesh uncoated copper clamshell grids.

The specimens were irradiated for ∼30 min before initiating a tilt series to limit anisotropic specimen thinning during image collection. During data collection, the illumination was held to near parallel beam conditions. In each sample, several electron microscopy images were captured using Hitachi HT7800 randomly, all at the same magnification. The mitochondrial length and form factor were measured using the ImageJ Fiji area and perimeter tools. To avoid bias, all the mitochondria in an image were measured. A total of at least 3 experiments were performed each time.

### Western Blot Assays

Proteins were extracted by homogenizing the cells or tissue in tissue lysate buffer (50 m m HEPES, pH 7.5, 150 m m NaCl, 1 m m MgCl2, 1 m m CaCl2, 10 m m NaF, 5 m m EDTA, 1% Triton, 2 m m sodium orthovanadate, and Roche cocktail protease inhibitor tablet). Protein samples (20 μg) were subjected to SDS PAGE, electro-transferred on a polyvinylidene fluoride membrane, then probed with primary antibodies (1:1000) targeting BBS3, DRP1, or phospho-DRP1 followed by a secondary anti-rabbit antibody (1:10 000). The protein membrane was striped to probe for β-actin, which was used to normalize loading. Protein expression was visualized with ECL detection kit (GE healthcare) and imaged with Sapphire Biomolecular Imager from Azure Biosystems.

### Data Analysis

The data are expressed as means ± SEM. Data were analyzed using *t-*test or 2-way analysis of variance (ANOVA) with repeated measures. When ANOVA reached significance, a post-hoc comparison was made using Fisher’s test or Tukey’s test. GraphPad PRISM 9.1.0 was used for statistical analysis. A *P* < .05 value was considered statistically significant.

## Results

### Inducible Loss of the *Bbs1* Gene in POMC Neurons Causes Obesity

To investigate the metabolic consequences of adult-onset disruption of POMC neuron BBSome, we generated mice that enable selective inducible deletion of the *Bbs1* gene in POMC neurons by breeding mice expressing tamoxifen-inducible Cre in POMC neurons (POMC^CreERT2^) with Bbs1^fl/fl^ mice. To validate Cre recombinase in POMC neurons, POMC^CreERT2^ mice were crossed with a reporter mouse model expressing fluorescent tdTomato (ROSA), in a Cre-dependent manner. First, we confirmed the Cre-mediated recombination by assessing tdTomato fluorescent protein expression 2 wk after POMC^CreERT2^/ROSA mice were treated with tamoxifen (Figure S1A and B). It should be noted that in the forebrain, tdTomato fluorescent protein was expressed almost exclusively in the arcuate nucleus of the hypothalamus, which is consistent with the well-known fact that the vast majority of POMC neurons are located in this nucleus.^31^ Next, we verified that tdTomato fluorescent protein mostly co-localized with POMC (Figure S1C) confirming the specificity of POMC^CreERT2^ mice. We also used single cell RNA PCR to confirm POMC neuron-specific loss of *Bbs1* gene expression in POMC^CreERT2^/Bbs1^fl/fl^/ROSA mice (Figure S1D) validating our strategy and mouse model.

**Figure 1.**
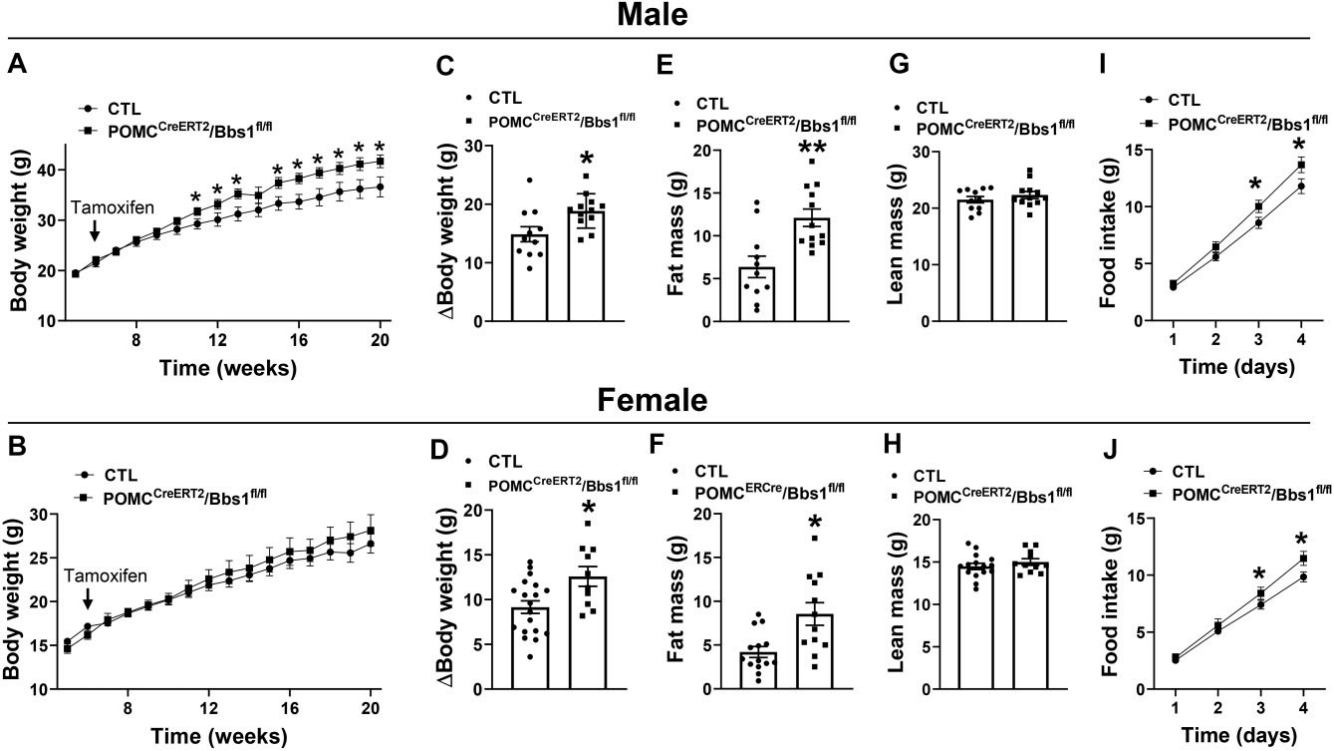
Proopiomelanocortin neuron-specific inducible *Bbs1* gene deletion increases body weight. (**A-B**) Effect of tamoxifen treatment (at 6 wk of age) on body weight of male (**A**) and female (**B**) POMC^CreERT2^/Bbs1^fl/fl^ mice and littermate controls (CTL). (**C-D**) Change in body weight (at 20 wk relative to 6 wk of age) in males (**C**) and females (**D**). (**E-F**) Fat mass in males (**E**) and females (**F**) at 20 wk of age. (**G**-**H**) Lean mass in males (**G**) and females (**H**) at 20 wk of age. (**I-J**) Cumulative 5 d food intake of males (**I**, *n* = 10-12 animals/group) and females (**J**, *n* = 12-15 animals/group) at 21 wk of age. *P < .05 and **P < .01 versus CTL.

To determine the consequence of adult-onset *Bbs1* gene deletion in POMC neurons, we assessed the effect of tamoxifen treatment on body weight. There was no difference in body weight between POMC^CreERT2^/Bbs1^fl/fl^ mice and littermate controls before and during administration of tamoxifen, at 6 wk of age (Figure 1A and B). However, 4 wk after tamoxifen (10 wk of age), body weight of male mice began to diverge with POMC^CreERT2^/Bbs1^fl/fl^ mice gaining more weight than the controls, with POMC^CreERT2^/Bbs1^fl/fl^ mice weighing about 4.4 g more than the controls at 20 wk of age (Figure 1A and C). Body weight of female POMC^CreERT2^/Bbs1^fl/fl^ mice began to diverge 6 wk after tamoxifen treatment (12 wk of age), but there was no statistical difference compared to controls (Figure 1B). Nonetheless, female POMC^CreERT2^/Bbs1^fl/fl^ mice gained significantly more weight than the controls at 20 wk of age (Figure 1D).

It should be noted that no significant difference in body weight was observed in male POMC^CreERT2^/Bbs1^fl/fl^ mice treated with corn oil relative to control animals treated with tamoxifen (Figure S2A) indicating that the excess weight gain in tamoxifen treated POMC^CreERT2^/Bbs1^fl/fl^ mice is not due to the presence of the POMC^CreERT2^ transgene.

The increase in body weight in POMC^CreERT2^/Bbs1^fl/fl^ mice was due to an increase in adiposity as indicated by the higher fat mass (Figure 1E and F) and weight of individual fat pads including brown adipose tissue and inguinal, gonadal, peri-renal white adipose tissues (Figure S2B and C). Liver mass was also significantly elevated (*P *< .05) in POMC^CreERT2^/Bbs1^fl/fl^ mice relative to controls (females: 1.16 ± 0.04 versus 1.09 ± 0.03 g, males: 1.98 ± 0.12 versus 1.62 ± 0.07 g). However, there was no difference in lean mass between POMC^CreERT2^/Bbs1^fl/fl^ mice and controls (Figure 1G and H), which was further confirmed by the lack of difference in the weight of the kidneys (Figure S2B and C). Consistent with the obesity phenotype, both male and female POMC^CreERT2^/Bbs1^fl/fl^ mice exhibited elevated food intake compared to their controls (Figure 1I and J).

### Inducible Loss of the *Bbs3* Gene in POMC Neurons Does Not Affect Body Weight

To determine whether inducible POMC neuron *Bbs3* gene deletion recapitulate the body weight phenotype induced by *Bbs1* gene deficiency, we generated a new mouse model that enables Cre-mediated specific deletion of the *Bbs3* gene (Bbs3^fl/fl^) by inserting 2 *lox*P sequences to flank exon 5 of the *Bbs3* gene (Figure S1E). Next, we crossed the Bbs3^fl/fl^ mice with the POMC^CreERT2^ mice to generate POMC^CreERT2^/Bbs3^fl/fl^ mice to allow inducible *Bbs3* gene specifically in POMC neurons (Figure S1D).

**Figure 2.**
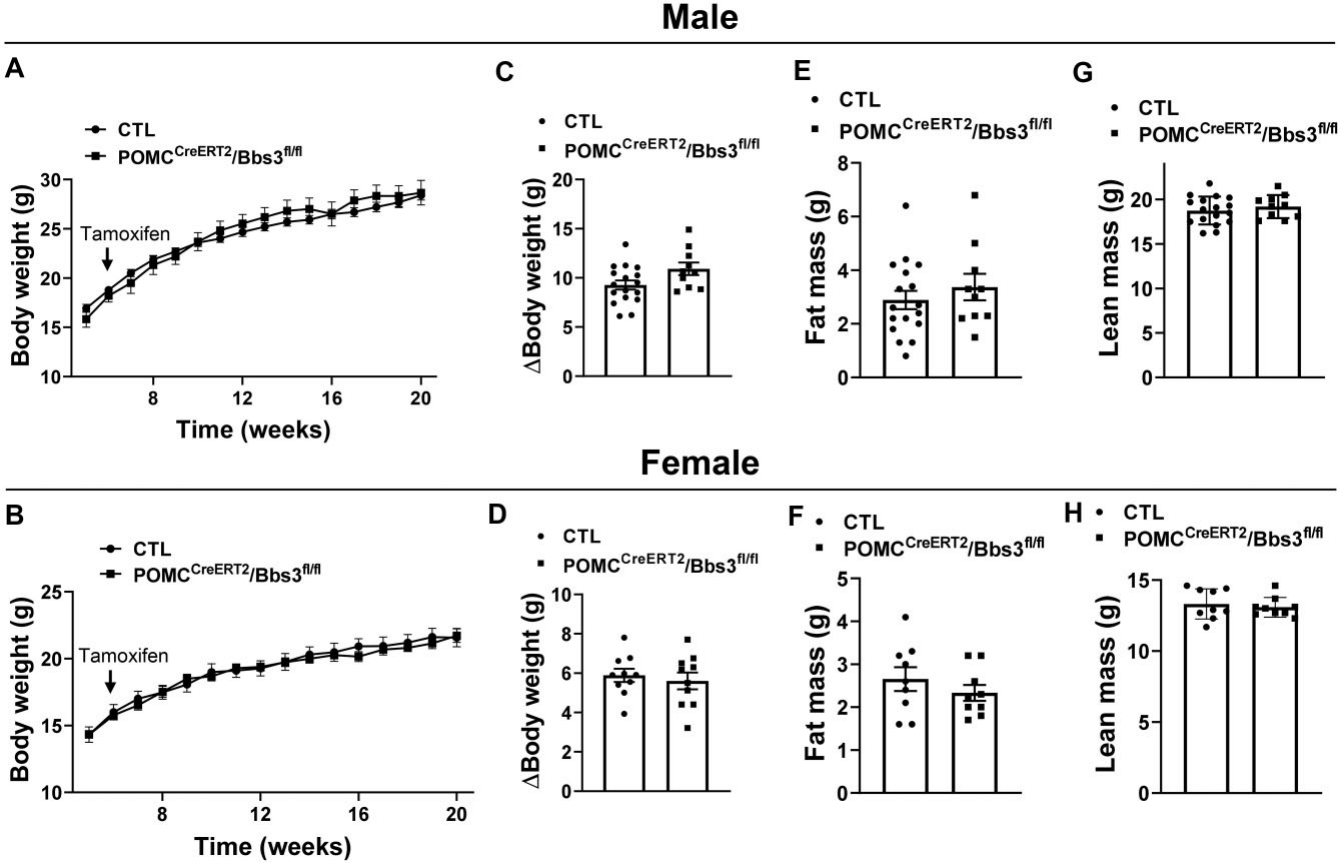
Proopiomelanocortin (POMC) neuron-specific inducible *Bbs3* gene deletion does not alter body weight. (**A-B**) Effect of tamoxifen treatment (at 6 wk of age) on body weight of male (**A**) and female (**B**) POMC^CreERT2^/Bbs3^fl/fl^ mice and littermate controls (CTL). (**C-D**) Change in body weight (at 20 wk relative to 6 wk of age) in males (**C**) and females (**D**). (**E-F**) Fat mass in males (**E**) and females (**F**) at 20 wk of age. (**G**-**H**) Lean mass in males (**G**) and females (**H**) at 20 wk of age.

We assessed how inducible deletion of the *Bbs3* gene in POMC neurons affect body weight and adiposity. However, tamoxifen treatment as above (for 5 d at 6 wk of age) caused no significant change in body weight in male and female POMC^CreERT2^/Bbs3^fl/fl^ mice relative to littermate controls (Figure 2A-D). Consistent with the unaltered body weight, fat mass and lean mass were comparable between POMC^CreERT2^/Bbs3^fl/fl^ mice and controls (Figure 2E-H). The weight of various fat pads and kidneys were also not different between the 2 groups of mice (Figure S3A and B).

### Effects of POMC Neuron *Bbs1* Versus *Bbs3* Gene Deletion on Glucose Handling and Insulin Sensitivity

**Figure 3.**
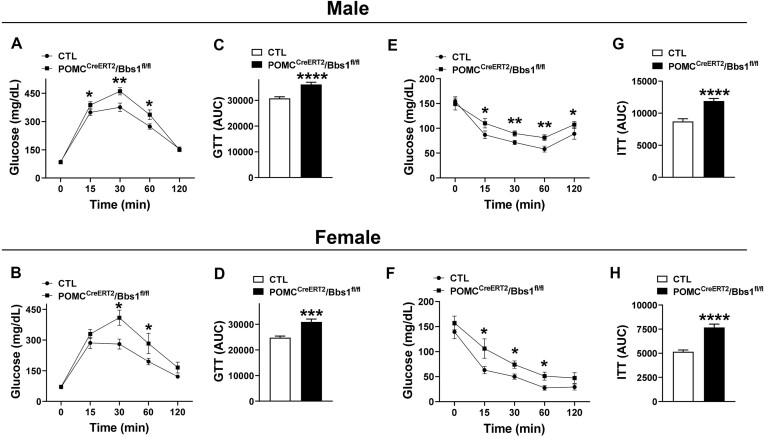
Proopiomelanocortin (POMC) neuron-specific inducible *Bbs1* gene deletion causes glucose intolerance and insulin resistance. (**A-B**) Glucose tolerance test in male (**A**, *n* = 10 animals/group) and female (**B**, *n* = 6/group) POMC^CreERT2^/Bbs1^fl/fl^ mice and littermate controls (CTL) at 21-22 wk of age. (**C-D**) Area under curve of glucose tolerance test (GTT) males (**C**) and females (**D**). (**E-F**) Insulin tolerance test in males (**E**, *n* = 9-10 animals/group) and females (**F**, *n* = 6-7 animals/group) at 21-22 wk of age. (**G-H**) Area under curve of GTT males (**G**) and females (**H**). *P < .05, **P < .01, ***P < .001, and ^****^P < .0001 versus CTL.

Given the importance of POMC neurons for the control of glucose metabolism and peripheral insulin sensitivity,^32, 33^ we asked whether inducible *Bbs* gene deletion affects glucose handling and insulin action. However, no significant change in fasting blood glucose was noted in POMC^CreERT2^/Bbs1^fl/fl^ mice (male: 89.1 ± 3.8 mg/dL, female: 76.1 ± 2.7 mg/dL) relative to controls (male: 82.9 ± 4.5 mg/dL, female: 66.7 ± 3.1 mg/dL). Next, we performed a glucose tolerance test, which revealed that both male and female POMC^CreERT2^/Bbs1^fl/fl^ mice have glucose intolerance. In control mice, following acute glucose challenge, blood glucose levels started to decline after peaking at 15-30 min and returned to near normal values after 2 h. However, in POMC^CreERT2^/Bbs1^fl/fl^ mice, after glucose administration, blood glucose levels peaked at 30 min and remained elevated at 60 min indicating reduced glucose excursion from blood (Figure 3A and B). Calculating the area under the curve confirmed the glucose intolerance in POMC^CreERT2^/Bbs1^fl/fl^ mice (Figure 3C and D).

To assess insulin’s ability to stimulate glucose mobilization, we performed an insulin tolerance test. In control mice, insulin treatment caused a robust decrease in blood glucose (Figure 3E-H). The blood glucose lowering effect of insulin was significantly attenuated in POMC^CreERT2^/Bbs1^fl/fl^ mice demonstrating the contribution of *Bbs1* gene in POMC neurons to insulin sensitivity.

**Figure 4.**
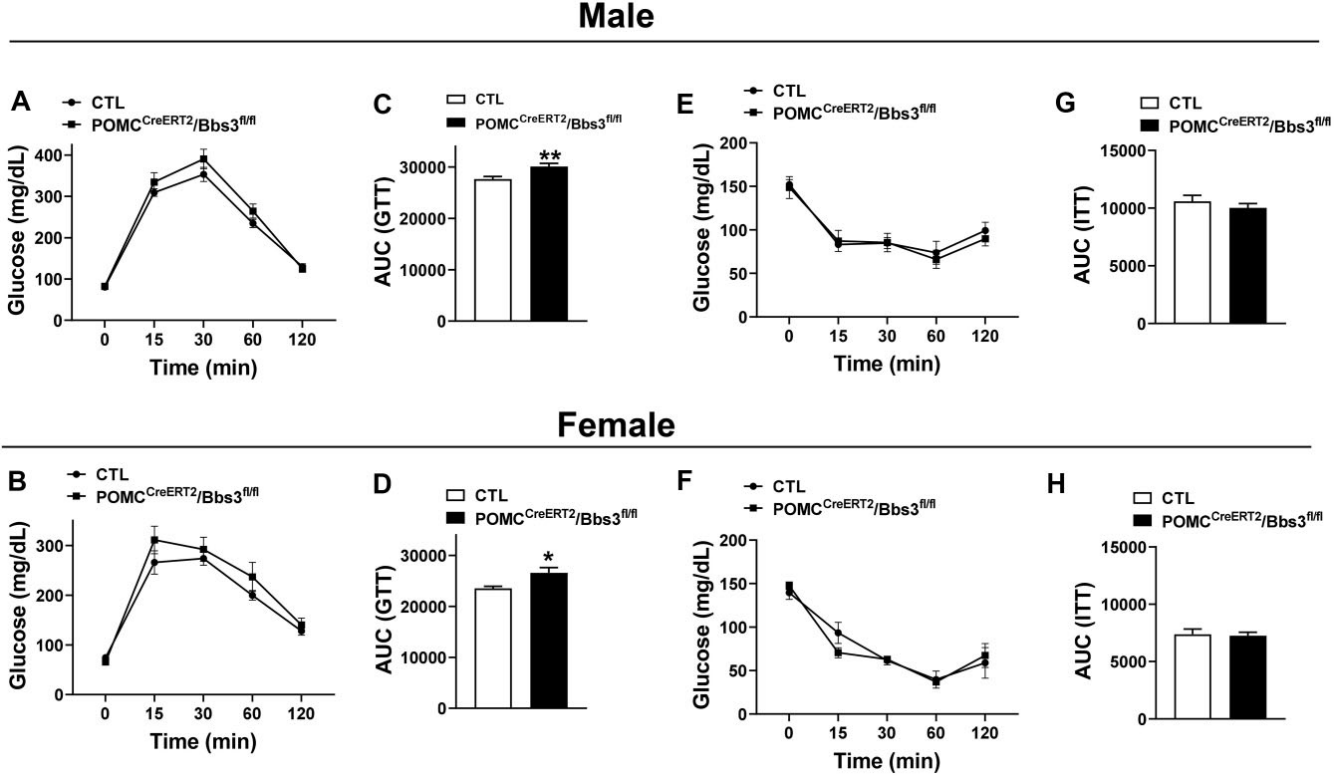
Proopiomelanocortin (POMC) neuron-specific inducible *Bbs3* gene deletion affect glucose tolerance. (**A-B**) Glucose tolerance test (GTT) in male (**A**, *n* = 8-12 animals/group) and female (**B**, *n* = 7-9 animals/group) POMC^CreERT2^/Bbs3^fl/fl^ mice and littermate controls (CTL) at 21-22 wk of age. (**C-D**) Area under curve of GTT males (**C**) and females (**D**). (**E-F**) Insulin tolerance test in males (**E**, *n* = 7-11 animals/group) and females (**F**, *n* = 6 animals/group) at 21-22 wk of age. (**G-H**) Area under curve of GTT males (**G**) and females (**H**). *P < .05 and **P < .01 versus CTL.

Fasting blood glucose was not different in POMC^CreERT2^/Bbs3^fl/fl^ mice relative to controls (male: *P *= .3, female: *P *= .1). Interestingly and despite the lack of body weight phenotype, glucose tolerance test shows a trend toward impairment in both male and female POMC^CreERT2^/Bbs3^fl/fl^ mice when compared to littermate controls (Figure 4A and B). The glucose intolerance in POMC^CreERT2^/Bbs3^fl/fl^ mice was more evident when calculating the area under the curve (Figure 4C and D). However, insulin tolerance test revealed no difference in insulin sensitivity between POMC^CreERT2^/Bbs3^fl/fl^ mice and controls (Figure 4E-H). Thus, glucose handling and insulin sensitivity are differentially affected by POMC neuron deletion of the *Bbs1* gene versus *Bbs3* gene.

### Validation of *BBS1^−/−^* and *BBS3^−/−^* RPE1 Cells

To understand the molecular mechanisms underlying differential metabolic effects induced by loss of *BBS1* gene versus *BBS3* gene in POMC neurons, we used CRISPR-Cas9 technology to generate RPE1 cells that lack these genes individually. We confirmed the absence of BBS3 expression in *BBS3^−/−^* cells by Western blot (Figure S4). However, the lack of specific BBS1 antibodies precluded us from confirming the loss of this protein in *BBS1^−/−^* cells. Nonetheless, we found that the endogenous BBS2 protein lost its ciliary localization in both *BBS1^−/−^* and *BBS3^−/−^* cells (Figures S5A and S6A-B) indicating that the trafficking of the BBSome to cilia is disrupted. Importantly, ciliary localization of the endogenous BBS2 was restored in *BBS1^−/−^* cells infected with AAV-*BBS1* (Figure S6C) confirming that loss of the endogenous BBS2 protein in cilia of *BBS1^−/−^* cells is due to *BBS1* gene deficiency. We further observed that BBS3 protein, which is absent in *BBS3^−/−^* cells locate throughout cilia in control cells, whereas it appears as punctate in *BBS1^−/−^* cells, localizing at the base of the cilium (Figure S5B-C). Co-staining with γ-tubulin, a basal body marker, revealed that in *BBS1^−/−^* cells, BBS3 did not colocalize with γ-tubulin, but was adjacent to it, inside the cilium (Figure S5D), likely in the transition zone. Thus, BBS1 deficiency disrupts ciliary localization of BBS3.

### Loss of BBS1, But Not BBS3, Alters Trafficking of Receptors That Regulate Metabolic Function

**Figure 5.**
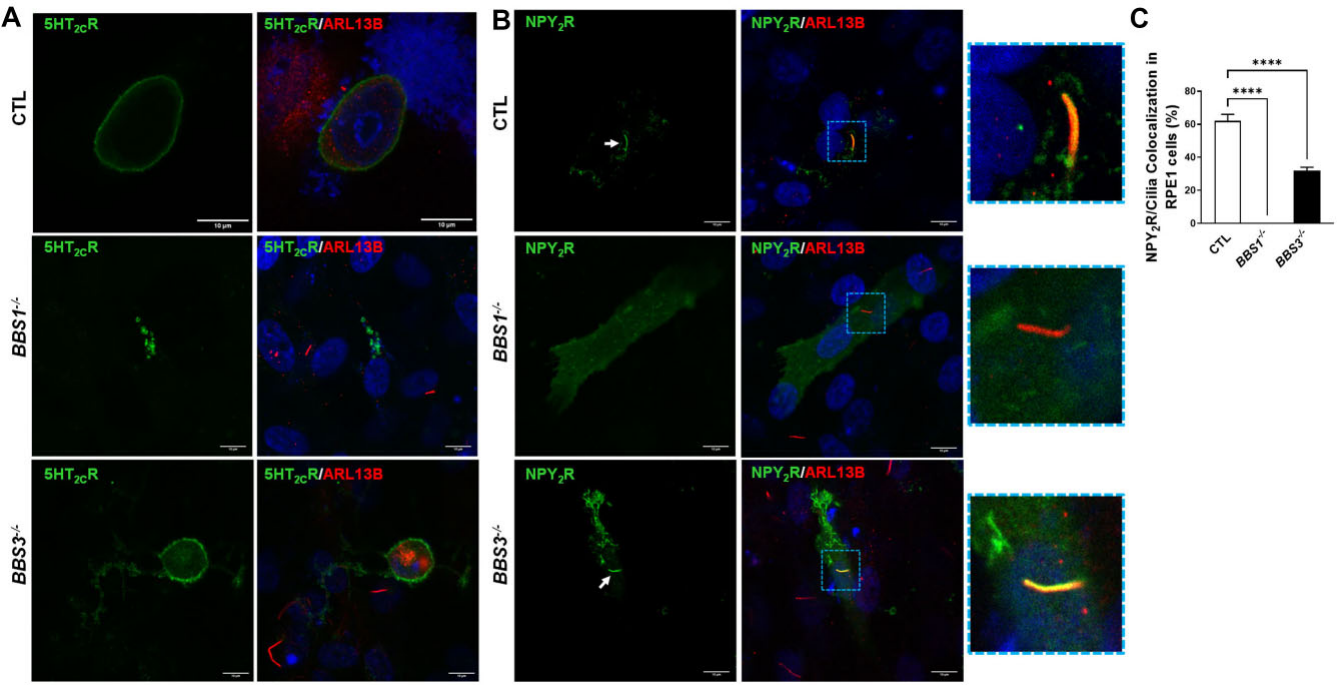
Effect of *BBS1* and *BBS3* gene ablation on the trafficking of key metabolic receptors. (**A**) Representative images of 5HT_2C_R and cilia (ARL13B) in control RPE1 (CTL), RPE1-*BBS1^−/−^*, and RPE1-*BBS3*^−/−^ cells (results are representative of 3 separate experiments). (**B**) Representative images of NPY_2_R and cilia of CTL, RPE1-*BBS1^−/−^*, and RPE1-*BBS3*^−/−^ cells. (**C**) Quantification of NPY_2_R located in cilia of CTL, RPE1-*BBS1^−/−^*, and RPE1-*BBS3*^−/−^ cells (3 separate experiments with 255-346 cells/experiment). ^****^P < .0001. Scale bar: 10 μm.

Evidence implicating BBS proteins in the trafficking of receptors involved in control of energy homeostasis, such as 5-HT_2C_R and NPY_2_R, led us to assess how deletion of *BBS1* and *BBS3* genes affect localization of these receptors. Consistent with our previous report,^16^ the 5-HT_2C_R locates predominantly in the plasma membrane of control cells with no localization in cilia (Figure 5A). Notably, the plasma membrane localization of 5HT_2C_R was altered in *BBS1^−/−^*, but not *BBS3^−/−^* cells. In *BBS1^−/−^* cells, the 5HT_2C_R appears stuck in the cytoplasm, likely in the late endosome.^16^ Next, we analyzed the effect of *BBS1* versus *BBS3* gene deletion on NPY_2_R trafficking, which predominantly localizes to the ciliary membrane.^16, 17^ In control cells, significant amount of NPY_2_R (62% ± 4%) localized in cilia (Figure 5B and C). Notably, this co-localization was substantially reduced in *BBS3*^−/−^ cells and absent in *BBS1*^−/−^ cells.

**Figure 6.**
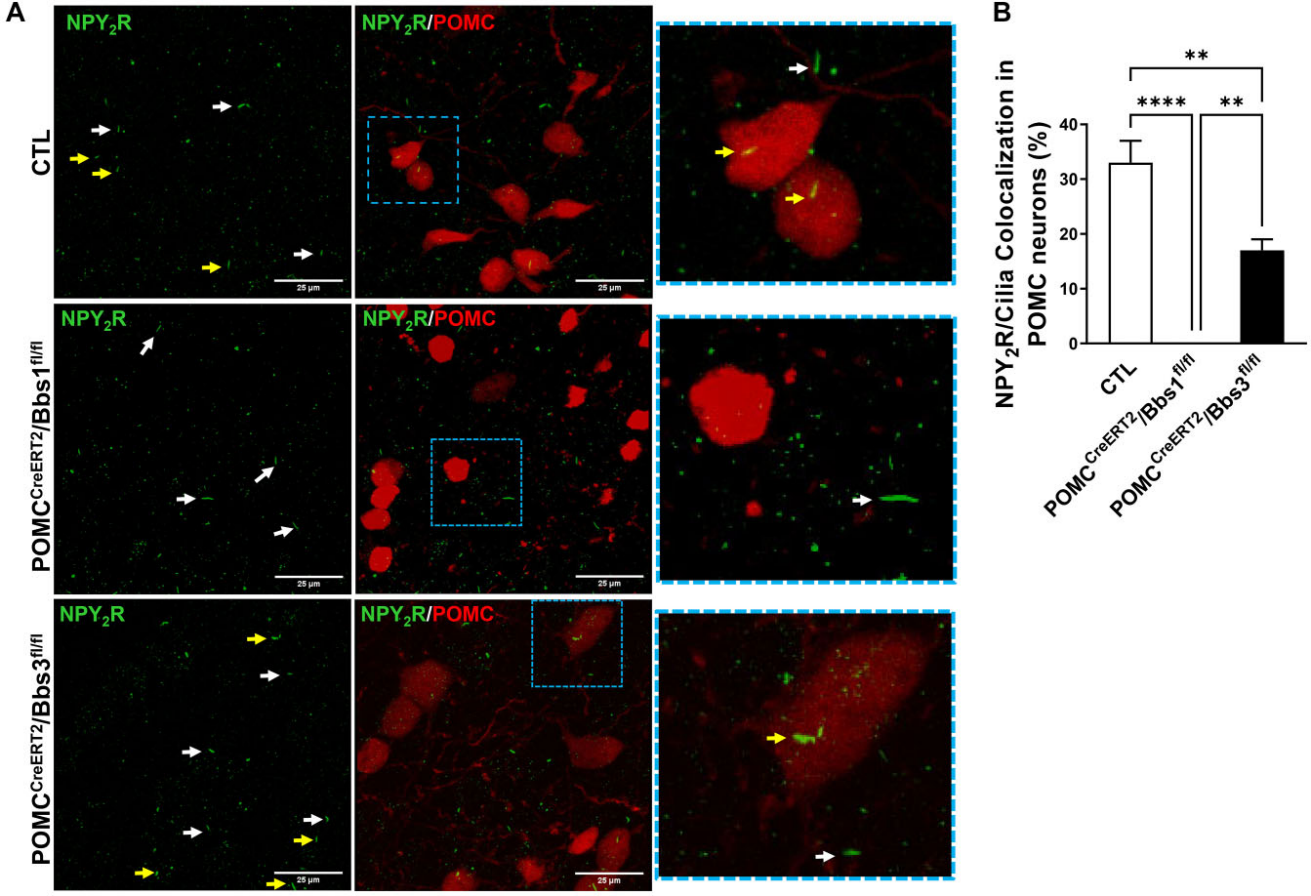
Effect of *Bbs1* and *Bbs3* gene deletion on ciliary localization of NPY_2_R in proopiomelanocortin (POMC) neurons. (**A**) Representative images of NPY_2_R in POMC neurons (tdTomato positive) and non-POMC cells (tdTomato negative) of the hypothalamic arcuate nucleus of control (CTL), POMC^CreERT2^/Bbs1^fl/fl^, and POMC^CreERT2^/Bbs3^fl/fl^ mice. Yellow arrows point to cilia of POMC neurons and white arrows point to cilia of non-POMC cells. (**B**) Quantification of NPY_2_R located in POMC neuron cilia of CTL, POMC^CreERT2^/Bbs1^fl/fl^, and POMC^CreERT2^/Bbs3^fl/fl^ mice (4 mice/group were used with 23-46 cells/cilia analyzed in each section of at least 4 sections per mouse). **P < .01 and ^****^P < .0001. Scale bar: 25 μm.

We also investigated the NPY_2_R localization in POMC neurons of the arcuate nucleus of the hypothalamus in control, POMC^CreERT2^/Bbs1^fl/fl^ mice and POMC^CreERT2^/Bbs3^fl/fl^ mice. In control mice, 33% ± 4% of cilia of POMC neurons are equipped with NPY_2_R (Figure 6A and B). Interestingly, this NPY_2_R/cilia co-localization was significantly reduced in POMC neurons of POMC^CreERT2^/Bbs3^fl/fl^ mice and absent in POMC neurons of POMC^CreERT2^/Bbs1^fl/fl^ mice. It should be noted that ciliary localization of NPY_2_R in non-POMC cells was not different between control, POMC^CreERT2^/Bbs1^fl/fl^, and POMC^CreERT2^/Bbs3^fl/fl^ mice. In contrast, ciliary localization of NPY_2_R was disrupted in POMC neurons of POMC^CreERT2^/Bbs1^fl/fl^ mice. Together, these data indicate that BBS1, but not BBS3, is required for the trafficking of key metabolic receptors to plasma membrane and cilia.

### Absence of BBS1, But Not BBS3, Affects Mitochondria Dynamics

**Figure 7.**
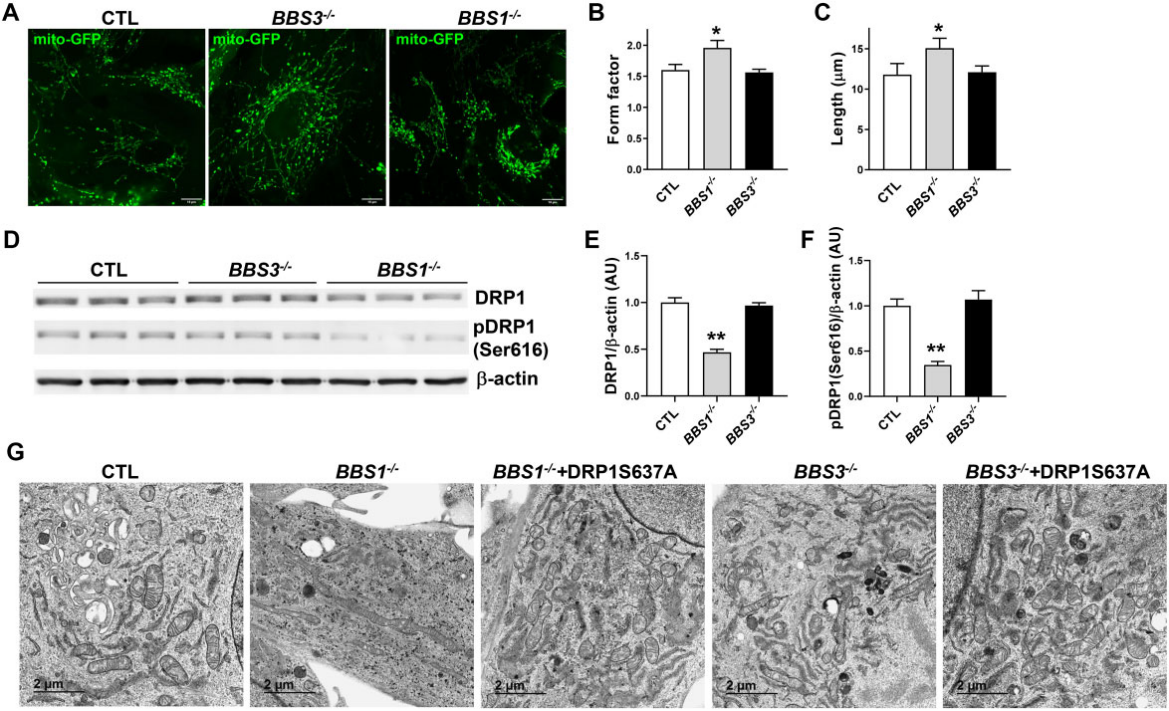
Differential effect of *BBS1* and *BBS3* gene deficiency on mitochondria dynamic. (**A-C**) Representative images of mitochondria of RPE1 (CTL), RPE1-*BBS1^−/−^*, and RPE1-*BBS3^−/-^* cells (**A**) and related quantification of mitochondrial form factor (**B**) and length (**C**) (*n* = 3, 28-37 images/group). (**D-F**) Representative Western blots (**D**) and related quantification of DRP1 (**E**) and pDRP1(Ser616) (**F**) in CTL, RPE1-*BBS1^−/−^*, and RPE1-*BBS3^−/-^* cells (*n* = 9 duplicates/group). (**G**) Representative TEM images of mitochondria of CTL, RPE1-*BBS1^−/−^*, and RPE1-*BBS3^−/-^* cells with or without FIV-DRP1S637A (results are representative of at least 3 separate experiments). *P < .05 and **P < .0001 versus CTL. Scale bar: 10 (**A**) and 2 μm (**G**).

Next, we assessed whether the control of mitochondria by the BBSome depends on its ciliary function. Mitochondrial-targeted GFP-mediated analysis of mitochondrial morphology showed that *BBS1* gene deficiency caused mitochondria hyperfusion as indicated by the increased mitochondrial form factor and length in *BBS1*^−/−^ cells (Figure 7A-C), which is consistent with our previous findings.^22^ Strikingly, these changes in mitochondria were not recapitulated in *BBS3^−/−^* cells. The role of DRP1 in mediating mitochondrial alterations in BBS led us to measure DRP1 phosphorylation at Ser616 [pDRP1(Ser616)]. A significant decrease in pDRP1(Ser616) was detected in *Bbs1*^−/−^, but not *Bbs3^−/−^* cells (Figure 7D-F). Analysis of mitochondrial morphology with transmission electron microscopy (TEM) confirmed that *BBS1*^−/−^, but not *BBS3^−/−^* cells display elongated mitochondria (Figure 7G). Moreover, infecting *BBS1^−/−^* cells with a DRP1S637A mutant, a mimetic of the dephosphorylated state^34, 35^ that rescues the decrease in pDRP1(Ser616) phosphorylation^22^ reversed the changes in mitochondrial morphology in *BBS1*^−/−^ cells without affecting mitochondria in *BBS3^−/−^* cells. These findings demonstrate that BBSome regulation of mitochondria is independent of its ciliary function.

**Figure 8.**
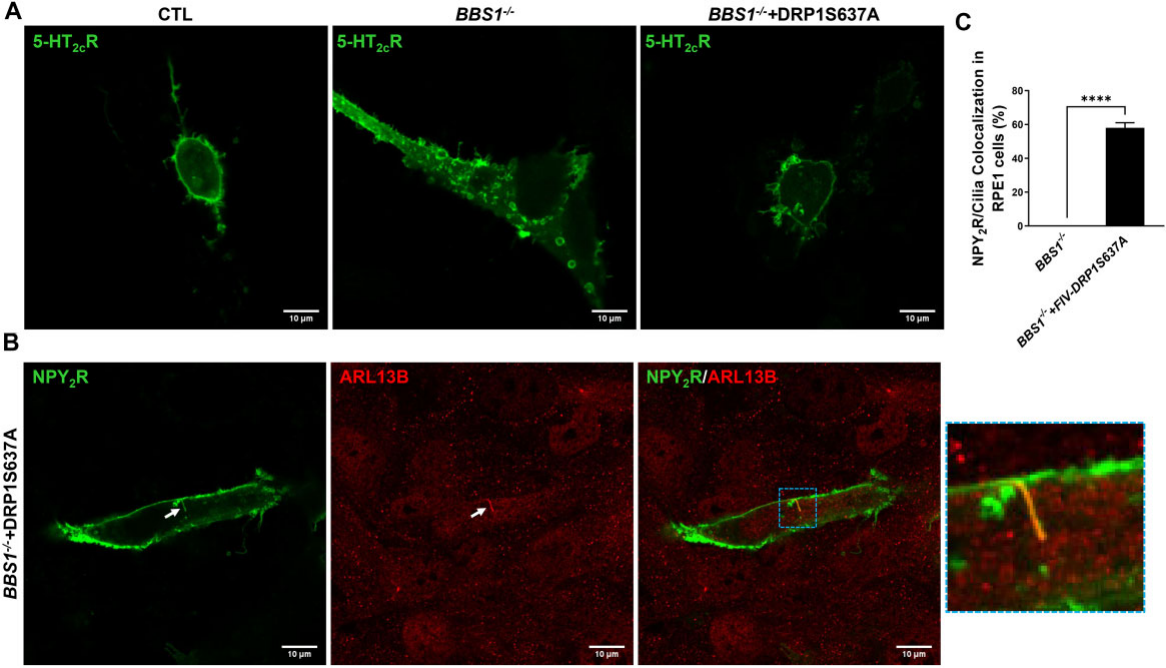
Rescuing the mitochondria defects restored the trafficking of metabolic receptors in BBS1-deficient cells. (**A**) Plasma membrane localization of 5HT_2C_R in RPE1-*BBS1^−/−^* cells treated with FIV-DRP1S637A, but not in RPE1-*BBS1*^−/−^ cells (results are representative of 4 separate experiments). (**B**) NPY_2_R located in cilia (ARL13B) of RPE1-*BBS1^−/−^* cells treated with FIV-DRP1S637A. (**C**) Quantification of NPY_2_R localization in cilia of RPE1-*BBS1^−/−^* cells treated with FIV-DRP1S637A versus FIV-control (4 separate experiments with 42-86 cells/cilia analyzed per experiment). Scale bar: 10 μm.

Finally, we asked whether the mitochondria defects may explain the receptor mistrafficking evoked by BBS1 deficiency. To test this, we examined the effect of restoring mitochondrial dynamic with DRP1S637A on 5-HT_2C_R and NPY_2_R localization in *BBS1^−/−^* cells. Interestingly, 5-HT_2C_R localization in the plasma membrane was restored in *BBS1^−/−^* cells infected with DRP1S637A (Figure 8A). Similarly, ciliary localization of NPY_2_R was substantially rescued by DRP1S637A in *BBS1^−/−^* cells (Figure 8B and C). These results point to mitochondrial defects as the underlying cause of receptor mislocalization associated with BBSome deficiency.

## Discussion

Proopiomelanocortin neurons are key regulators of energy homeostasis by controlling downstream neurocircuits that promote satiety, increase energy expenditure, and contribute to weight loss.^8^ Here, we demonstrate contrasting metabolic effects evoked by POMC neuron deletion of a key component of the BBSome, BBS1, versus BBS3, which mediate the BBSome trafficking to cilia. Loss of the *Bbs1* gene in POMC neurons triggered a significant increase in body weight and adiposity. In contrast, POMC neuron *Bbs3* gene deletion failed to affect body weight and fat mass. Moreover, POMC neuron *Bbs1* gene deletion caused glucose intolerance and insulin resistance, whereas loss of the *Bbs3* gene in these same neurons is associated with normal insulin sensitivity and mild glucose intolerance. Interestingly, disruption of the BBSome hindered the trafficking and localization of 5-HT_2C_R and NPY_2_R, 2 important receptors for energy balance. On the other hand, BBS3 deficiency did not interfere with plasma membrane localization of 5-HT_2C_R, but reduced the trafficking of NPY_2_R to cilia. Moreover, deficiency in the BBSome, but not BBS3, impaired mitochondria dynamics through reduction in DRP1 activity. Rescuing the diminished DRP1 activity restored the proper localization of 5-HT_2C_R and NPY_2_R in plasma membrane and cilia, respectively, in BBSome-deficient cells. These data highlight the importance of the BBSome in POMC neurons for control of energy and glucose balance while BBS3 appears minimally involved in glucose handling. Together, our findings indicate that POMC neuron BBSome regulation of energy homeostasis is not linked to its ciliary function.

The increase in body weight and adiposity by inducible *Bbs1* gene ablation in POMC neurons is in line with the effects of constitutive deletion of this gene in these neurons.^16^ This argues against the idea that BBS-associated obesity is caused by neurodevelopmental defects that arises from prenatal loss of *BBS* genes.^36^ It should be noted, however, that the weight gain and adiposity evoked by inducible *Bbs1* gene ablation in POMC neurons was less pronounced relative to those evoked by its constitutive deletion. This difference in weight gain and adiposity may reflect the more severe impact of BBSome disruption during embryonic stage versus adult-onset phase. Alternatively, this may be due to a broader loss of the *Bbs1* gene in mice bearing the constitutive POMC^Cre^. Indeed, constitutive POMC^Cre^ mice displayed Cre expression not only in POMC neurons, but also in other neuronal populations,^37^ which may have contributed to the pronounced obesity phenotype in POMC^Cre^ mice relative to POMC^CreET2^ mice. Irrespective of this issue, our data demonstrate that the BBSome in POMC neurons is required for energy balance.

The prevalence and severity of obesity in BBS3 patients is variable within and among families. For instance, Young et al. reported that most BBS3 patients of northern European descent displayed no or mild obesity phenotype,^38^ whereas most BBS3 patients of Arab-Bedouin and Iranian families were found to exhibit marked obesity.^39, 40^ We previously reported that the phenotype developed by global *Bbs3* null mice do not entirely mimic those observed in other BBS knockout mice.^26^ In particular, *Bbs3* null mice displayed minimal increase in body weight, adiposity, and plasma leptin. Here, we extend these findings by demonstrating that in contrast to *Bbs1* gene ablation, *Bbs3* gene deletion in POMC neurons failed to affect body weight and adiposity. These results indicate that increased body weight in *Bbs3* null mice is not due to loss of BBS3 function in POMC neurons. These findings also show that the contribution of the POMC neuron BBSome to energy homeostasis is independent of its interaction with BBS3 and by extension from its ciliary function. The notion that BBSome control of energy balance is not linked to cilia is further supported by the minimal or no effect on body weight and adiposity of loss of cilia (*Ift88* gene) in the leptin sensitive neurons, whereas *Bbs1* gene deletion in these same neurons causes frank obesity.^20, 41^ Rather, the BBSome appears to influence body weight through the regulation of the plasma localization of key receptors involved in energy homeostasis such as the 5-HT_2C_R.

Type 2 diabetes is a common observation in BBS patients.^42, 43^ Using BBS mice, we demonstrated that this phenotype is due to mis-trafficking of the insulin receptor.^28^ Our current investigation revealed the importance of *Bbs1* gene in POMC neurons for the regulation of glucose metabolism and insulin sensitivity as indicated by the glucose intolerance and insulin resistance observed in POMC^CreERT2^/Bbs1^fl/fl^ mice. Remarkably, inducible deletion of the *Bbs3* gene in POMC neurons caused slight impairment in glucose tolerance despite normal body weight and adiposity. This indicates that the glucose phenotype in BBS mice is independent of obesity. This is in line with our previous findings of insulin resistant in global BBS mice even when kept lean using caloric restriction.^28^ Our results add to the growing body of evidence pointing to the importance of POMC neurons in the regulation of glucose metabolism and insulin sensitivity. Previous studies have implicated various proteins and signaling pathways in POMC neurons in the control of peripheral glucose metabolism and insulin action.^32, 33^ We extend these findings by implicating BBS proteins in POMC neurons in glucose tolerance and insulin sensitivity. However, additional work is needed to understand how loss of BBS proteins in POMC neurons alters these parameters.

The differential body weight and metabolic phenotypes resulting from loss of BBS1 versus BBS3 seem to have its explanation in the distinct involvement of these 2 proteins in the handling of metabolic receptors. Consistent with previous findings,^16, 17^ we showed that BBS1 is required for proper localization of 5-HT_2C_R and NPY_2_R to plasma membrane and cilia, respectively, in cultured cells and POMC neurons. Importantly, we show that BBSome regulation of the trafficking of these receptors relate to mitochondria function as indicated by the restored 5-HT_2C_R and NPY_2_R localization following rescue of mitochondrial defects in *BBS1^−/−^* cells. On the other hand, loss of BBS3 did not affect the trafficking of 5-HT_2C_R nor the mitochondria dynamic, but reduced the ciliary localization of NPY_2_R. The dispensability of BBS3 for 5-HT_2C_R trafficking is consistent with the notion that BBSome transport of receptors to plasma membrane is independent of its ciliary function.^20^ However, the fact that ∼34% of NPY_2_R still traffic to cilia in absence of BBS3 is puzzling because BBS3 is involved in the recruitment and trafficking of the BBSome to cilia,^26^ which we confirmed by showing that loss of BBS3 alter the ciliary localization of the BBSome component BBS2. Of note, crystal structure analysis has demonstrated that BBS3 promote the BBSome entry into cilia through its binding to BBS1.^24^ This interaction is abolished by single point mutations in the BBS3-BBS1 interface preventing the import of BBSome into cilia. Mutations in BBS1 (e.g. M390R) also disrupted the interaction of the BBSome with BBS3.^24^ Thus, it is not clear how the BBSome mediates trafficking of NPY_2_R to cilia in absence of BBS3. One potential explanation is that in absence of BBS3, other proteins may mediate trafficking of the BBSome cargos such as the NPY_2_R to the ciliary membrane. For instance, tubby family protein TULP3 has been implicated in the ciliary localization of various G protein-coupled receptors.^44, 45^ However, future studies are warranted to test whether compensatory mechanisms overcome the loss of BBS3 and mediate the trafficking of NPY_2_R to cilia. Additional studies are also needed to determine whether loss of ciliary localization of NPY_2_R contribute to the metabolic defects evoked by BBSome deficiency in POMC neurons.

Our findings reinforce the notion that the BBSome is a key modulator of mitochondria dynamic and function by regulating the activity of DRP1, a core component of the canonical mitochondrial fission machinery in mammals. However, the exact mechanisms underlying BBSome regulation of DRP1 levels and activity are not fully understood. We previously demonstrated that DRP1 protein was significantly decreased in mitochondria isolated from BBS1-deficient cells,^22^ indicating that absence of the BBSome disrupts mitochondrial localization of DRP1, which may lead to its degradation decreasing its total and phosphorylated levels. Alternatively, dysregulation in DRP1 phosphorylation when the BBSome is absent^22^ may promote its degradation. More studies are necessary to test these possibilities.

Our findings provide additional evidence that point to a neuronal origin of the obesity associated with BBS. Specifically, our results show that defects in the first-order neurons (e.g. POMC neurons) as the underlying cause of obesity in BBS. Importantly, such defects can be bypassed by targeting downstream neurocircuits. Indeed, we showed that stimulation of MC4R cause a significant decrease in food intake and body weight in BBS mice.^21^ This observation has led to several clinical trials that demonstrated the efficacy of an MC4R agonist, Setmelanotide, as a treatment option for obesity in individuals with BBS.^46, 47^ Setmelanotide is now approved for the management of obesity in BBS patients, improving their health-related quality of life.^48^ This highlights the importance of understanding the molecular processes that underlie the obesity associated with BBS, which may also help decipher the mechanisms of polygenic common human obesity since variants of several *BBS* genes were found to increase susceptibility to obesity and type 2 diabetes in non-BBS individuals.^49–51^

## Supplementary Material

zqad070_Supplemental_File

## Data Availability

Data and materials may be requested by the corresponding author.
