# Supplementary material for: POMC Neuron BBSome Regulation of Body Weight is Independent of its Ciliary Function
Source: Function (Oxf). 2023 Dec 23;5(1):zqad070. doi: 10.1093/function/zqad070 (PMC10787280; doi:10.1093/function/zqad070)

**Online Supplement**

**POMC Neuron BBSome Regulation of Body Weight is Independent of its Ciliary Function**

Deng-Fu Guo^1,2^, Paul A. Williams^1^, Connor Laule^1^, Charles Seaby^3^, Qihong Zhang^3^, Val C Sheffield^3,4,5^ and Kamal Rahmouni^1,2,4,5,6,7^*

^1^Department of Neuroscience and Pharmacology, University of Iowa Carver College of Medicine, Iowa City, Iowa, United States of America

^2^Veterans Affairs Health Care System, Iowa City, Iowa, United States of America

^3^Department of Pediatrics, University of Iowa Carver College of Medicine, Iowa City, Iowa, United States of America

^4^Fraternal Order of Eagles Diabetes Research Center, University of Iowa Carver College of Medicine, Iowa City, Iowa, United States of America

^5^Iowa Neuroscience Institute, University of Iowa Carver College of Medicine, Iowa City, Iowa, United States of America

^6^Obesity Research and Education Initiative, University of Iowa Carver College of Medicine, Iowa City, Iowa, United States of America

^7^Department of Internal Medicine, University of Iowa Carver College of Medicine, Iowa City, Iowa, United States of America

**Short title:** POMC Neuron BBS Proteins

***Corresponding author:**

Kamal Rahmouni, Ph.D.

Department of Neuroscience and Pharmacology

University of Iowa Carver College of Medicine

Iowa City, IA, 52242, USA

e-mail: kamal-rahmouni@uiowa.edu

###### Tel: 319 353 5256

Fax: 319 353 5350

**
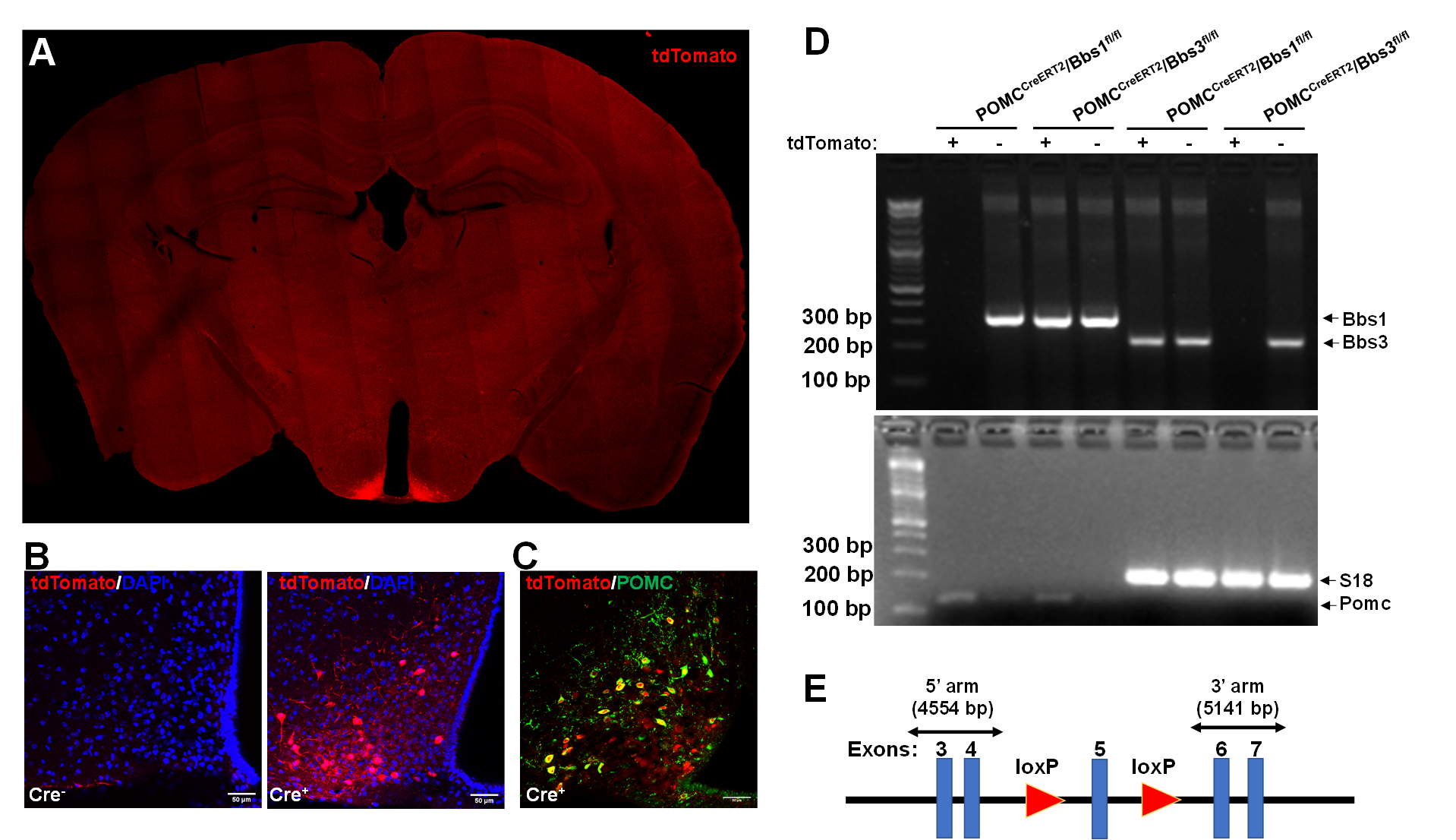
**

**Supplemental Figure 1.** Validation of Cre recombinase activity in POMC^CreERT2^ mice. **A**, Representative image showing restricted tdTomato fluorescent protein expression to the arcuate nucleus of the hypothalamus of POMC^CreERT2^/ROSA mice two weeks after tamoxifen administration. **B**, Presence of tdTomato in the arcuate nucleus in Cre positive (POMC^CreERT2^) mice, but not in control Cre negative mice. **C**, Co-localization (yellow) of tdTomato (red) with POMC (green) in the arcuate nucleus of POMC^CreERT2^/ROSA mice. **D**, *Bbs1* and *Bbs3* gene expression is detected in tdTomato negative cells (which do not express *Pomc*), but not in tdTomato positive neurons (which express *Pomc*) of POMC^CreERT2^/Bbs1^fl/fl^/ROSA and POMC^CreERT2^/Bbs3^fl/fl^/ROSA mice, respectively. S18 expression was used as loading control. **E**, Schematic representation of the floxed allele of the *Bbs3* gene construct used to generate Bbs3^fl/fl^ mice.

**
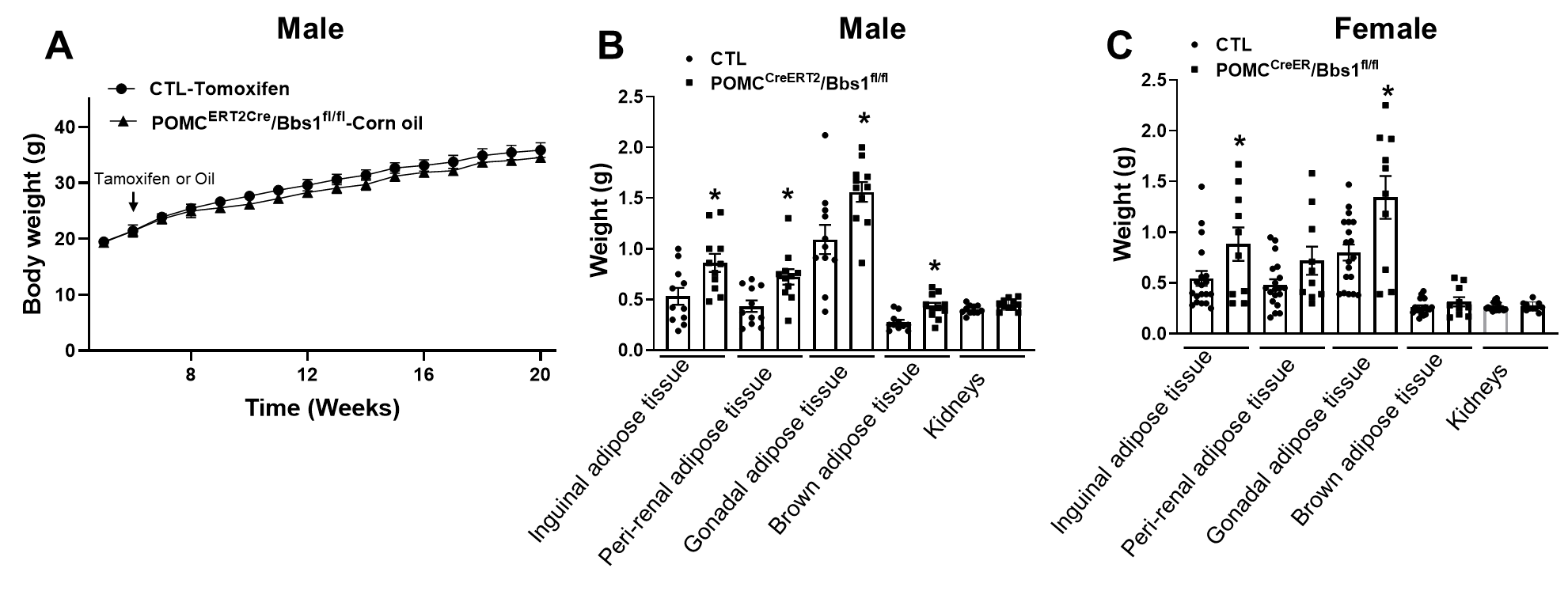
Supplemental Figure 2.** Weight gain in POMC^CreERT2^/Bbs1^fl/fl^ mice is not due to the POMC^CreERT2^ transgene and is associated with an increase in adiposity. **A**, Comparison of body weight between POMC^CreERT2^/Bbs1^fl/fl^ mice treated with vehicle (Corn oil) and littermate controls (CTL) treated with tamoxifen. Note that the data of the tamoxifen treated CTL group is the same as in Figure 1A. **B-C**, Weight of different fat pads and kidneys of male (**B**) and female (**C**) POMC^ERCre^/Bbs1^fl/fl^ mice and littermate controls (CTL). *P<0.05 vs controls.

**
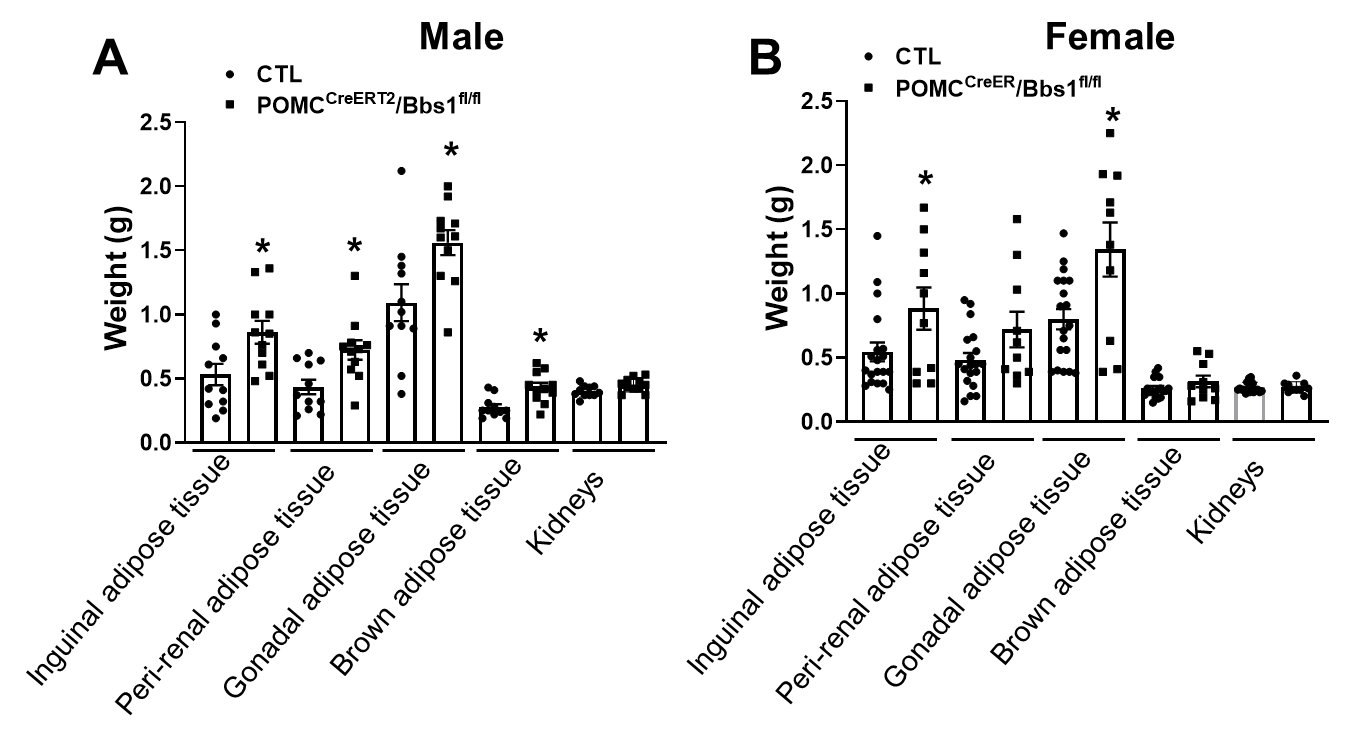
**

**Supplemental Figure 3**. POMC neuron-specific inducible *Bbs3* gene deletion does not affect adiposity. **A-B**, Weight of different fat pads and kidneys of male (**A**) and female (**B**) POMC^ERT2Cre^/Bbs3^fl/fl^ mice and littermate controls (CTL).

**
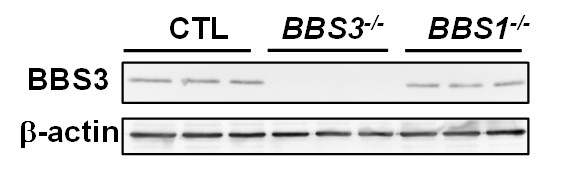
**

**Supplemental Figure 4.** Representative Western blots confirming the deletion of BBS3 protein in RPE1-*BBS3^-/-^* cells. On the other hand, BBS3 expression is not altered in *BBS1^-/-^* cells.

**Supplemental Figure 5**. Loss of ciliary localization of endogenous BBS2 in both RPE1-*BBS1^-/-^* and RPE1-*BBS3^-/-^* cells. **A**, Endogenous BBS2 protein localize in the cilia (acetylated α-tubulin) in RPE1 cells, but not in RPE1-*BBS1^-/-^* and RPE1-*BBS3^-/-^* cells. **B**, Decreased ciliary localization of the endogenous BBS3 protein in RPE1-*BBS1^-/-^* cells compared to control cells. Note that BBS3 protein is not detectable in RPE1-*BBS3^-/-^* cells. **C-D**, In RPE1-*BBS1^-/-^* cells, the endogenous BBS3 protein localizes at the base of cilia (C) near γ-tubulin, a marker of the basal body (D). Scale bar: 10 μm.

**
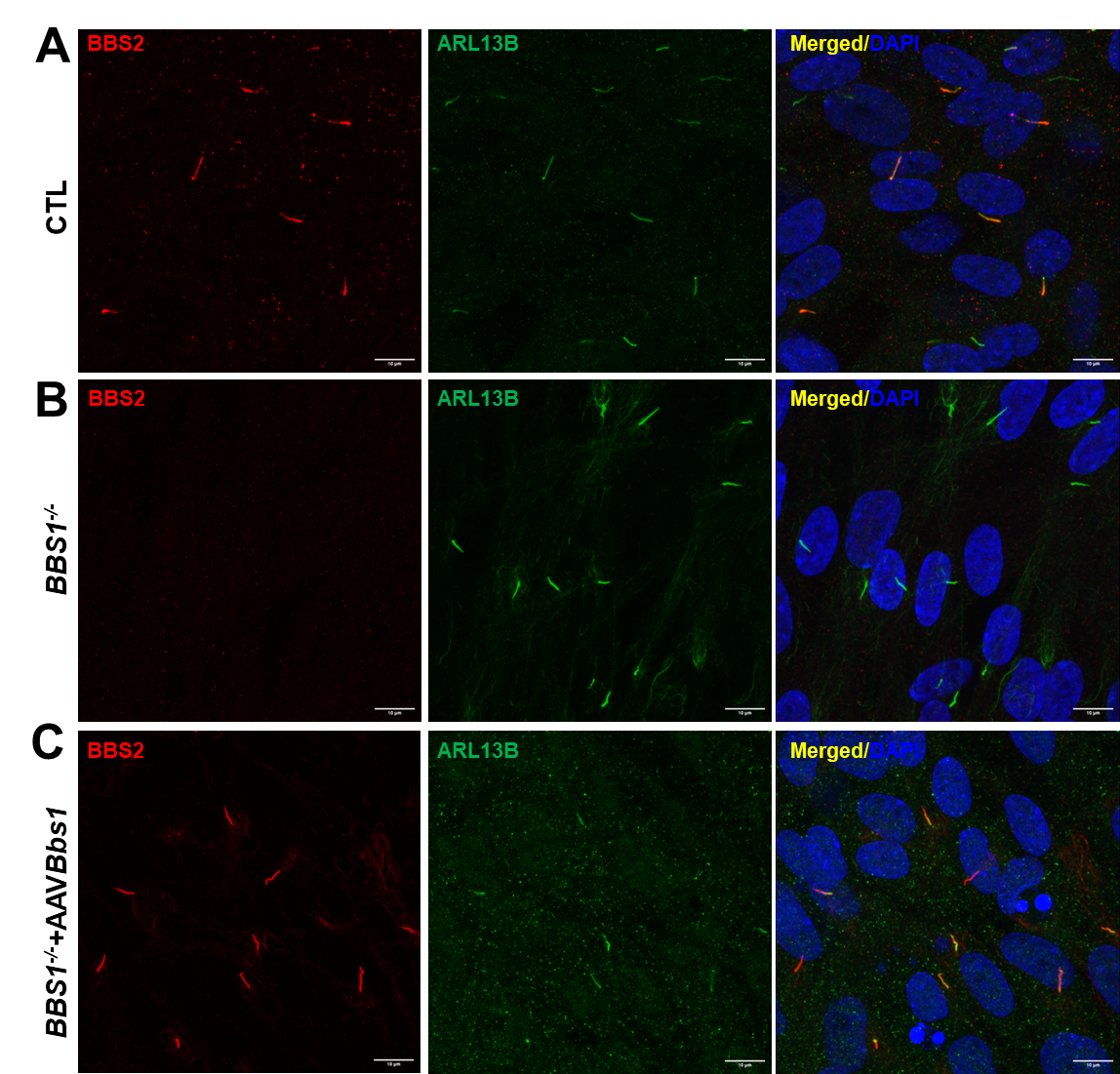
**

**Supplemental Figure 6**. Rescue of ciliary localization of endogenous BBS2 in RPE1-*BBS1^-/-^* cells infected with AAV-*Bbs1*. **A** and **B**, Representative images showing BBS2 protein localized in cilia (ARL13b) of control RPE1 cells, but not in the RPE1-*BBS1^-/-^* cells. **C**, Restoration of ciliary localization of BBS2 in RPE1-*BBS1^-/-^* cells infected with AAV-*Bbs1*. Scale bar: 10 μm.

**Supplemental Table 1: Primer sequences used for genotyping and RT-PCR.**


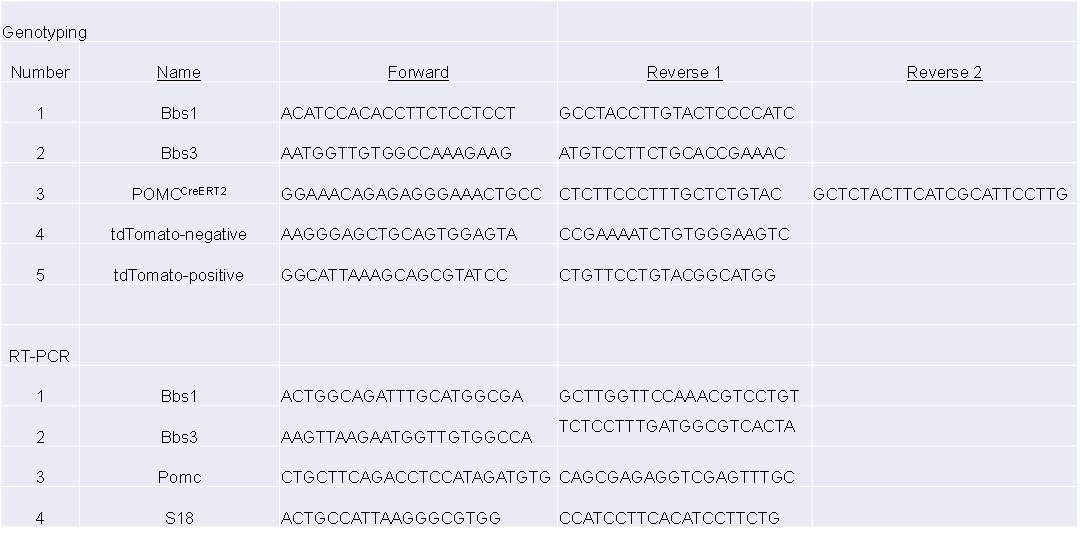

Supplement: zqad070_Supplemental_File [file zqad070_supplemental_file.docx]
